# Role of Extracellular Vesicles in Thyroid Physiology and Diseases: Implications for Diagnosis and Treatment

**DOI:** 10.3390/biomedicines10102585

**Published:** 2022-10-15

**Authors:** Ophélie Delcorte, Jonathan Degosserie, Christophe E. Pierreux

**Affiliations:** 1 CELL Unit, de Duve Institute, Université Catholique de Louvain, 1200 Brussels, Belgium; 2 Department of Laboratory Medicine, Molecular Diagnostic Center, CHU UCL Namur, 5530 Yvoir, Belgium

**Keywords:** thyroid, thyroid cancer, graves, Hashimoto, extracellular vesicles, exosomes, biomarker, therapy

## Abstract

Extracellular vesicles are spherical subcellular structures delimited by a lipid bilayer and released by most cells in the human body. They are loaded with a myriad of molecules (i.e., nucleic acids and proteins) depending on their cell of origin and provide the ability to transmit a message to surrounding or distant target cells. In several organs, including the thyroid, abundant recent literature reports that extracellular vesicles are responsible for intercellular communication in physiological and pathological processes, and that their utilization as a potential biomarker of pathological states (i.e., cancer, autoimmune diseases) or as therapeutic delivery vehicles promise clinical options. In this review, we present the current knowledge and understanding regarding the role of extracellular vesicles in developing thyroid diseases and diagnosis.

## 1. Thyroid Physiology and Thyroid Diseases

**Figure 1:**
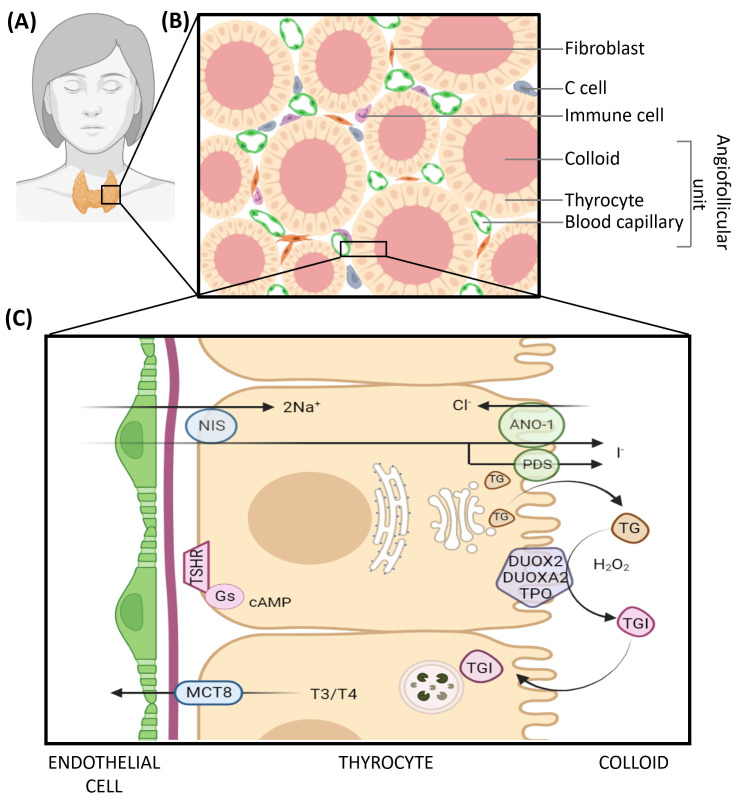
Histology and function of the healthy thyroid gland. (**A**) Anatomical localization of the gland. (**B**) Schematic representation of the thyroid parenchyma. (**C**) Oversimplified representation of thyroid hormone synthesis with major actors. The synthesis of thyroid hormones, namely thyroxine or tetra-iodothyronine (T4) and tri-iodothyronine (T3), begins in thyrocytes with the expression of thyroglobulin (TG). TG is glycosylated in the Golgi and secreted by exocytosis in the follicular lumen. Concomitantly, thyrocytes captures iodide (I^−^) from the bloodstream via the sodium/iodine symporter (NIS) located at their basolateral pole. Cytoplasmic iodide is then transferred across the apical membrane into the lumen via Anoctamin-1 (ANO-1) and Pendrin (PDS). Within the lumen, iodide is oxidized to di-iodine (I_2_) and serves as a substrate for thyroglobulin iodination on tyrosine residues. TPO catalyzes the covalent binding of mono-iodide or di-iodide to tyrosine residues of thyroglobulin (MIT or DIT) and different coupling reactions between modified residues thereby generating iodinated thyroglobulin (TGI). Those reactions require the presence of H_2_0_2_ generated by DUOX2 and DUOXA2 proteins. Upon TSH binding to its receptor at the basal membrane, colloid droplets are endocytosed (among other events), and endosomes fuse with lysosomes. TGI is hydrolyzed by lysosomal proteases, and the released T3/T4 hormones are transported in the cytoplasm. They are finally exported from the thyrocyte to the extracellular milieu via the monocarboxylate transporter MCT8; they cross the basal lamina and reach the blood flow through the fenestrated capillaries.

The thyroid gland is an endocrine organ responsible for the production, storage and release of thyroid hormones (T3, T4), thanks to thyrocytes (Figure 1), and for the production of calcitonin, thanks to C cells. Three essential characteristics are required for normal and adequate thyroid function and T3/T4 production: (i) thyroid epithelial cells or thyrocytes display a strict apico-basal polarity delimiting two membranous domains equipped with specific enzymes, receptors and transporters, (ii) polarized thyrocytes form a multitude of epithelial monolayers, with each organized as a closed spherical structure, the follicle, and containing the colloid, a reservoir of thyroid hormone precursors, and (iii) follicles are intimately associated with blood vessels allowing molecular exchanges between these two compartments [1]. Thyroid hormone production and, indirectly, the structure of angiofollicular units are regulated by the Thyroid Stimulating Hormone (TSH) produced by the anterior pituitary upon stimulation by thyroliberin (TRH) secreted by the hypothalamus when the T3 and T4 blood levels decrease. The binding of TSH to its receptor on the basolateral membrane domain of thyrocyte induces several molecular changes, such as increased expression level of thyroglobulin, as well as enzymes and transporters involved in TH synthesis (NIS, TPO), their translocation at the plasma membrane and the increased rate of colloid endocytosis and digestion in lysosomes. The vascular network covering each follicle is abundant and can evolve depending on the follicular activity, the increased surface upon iodine deficiency, or reduced T3/T4 blood levels and vice versa. The alteration of the follicular structure or its regulation causes defects in thyroid hormone secretion, such as hypo- or hyperthyroidism. These defects may originate in autoimmune thyroid diseases (AITD), iodine deficiency, or thyroid cancer (TC) and sometimes result in goiter development.

Goiter is referred to as the abnormal enlargement of the thyroid, mainly due to hormonal imbalance. Goiters can be associated with hypo- or hyperthyroidism, depending on its cause. Some goiters are diffuse, while others present multiple nodules (MNG). Thyroid nodules are caused by hyperplasia of thyrocytes, creating lumps inside or at the surface of the thyroid gland. The presence of unpalpable small thyroid nodules is found in the majority (60–70%) of the adult population, as documented by high-resolution ultrasonography. However, those nodules are almost always benign (85–90%) [2].

Thyroid cancer is the most common endocrine malignancy, accounting for 3–4% of all cancers diagnosed annually, and it affects three times more women than men. Until recently, thyroid cancer was the most rapidly increasing cancer diagnosed in developed countries. Different types of thyroid cancers are distinguished based on their differentiation stage, histology, and causal mutation. They are subdivided into four types: differentiated thyroid carcinoma (DTC), including papillary, follicular and Hürthle cell, medullary carcinoma (MTC), anaplastic carcinoma (ATC) and poorly differentiated TC [3]. Most DTC originate from the follicular cells that remain differentiated, progress slowly and have a good prognosis. Papillary thyroid cancer (PTC) is the most common, diagnosed in more than 80% of TC cases. Even though PTCs grow slowly, they can dedifferentiate and acquire important invasive capabilities, leading to metastasis (often in the neck lymph nodes), recurrence, and resistance to radioactive iodine treatments. Follicular thyroid cancer (FTC) is the second most common subtype, representing 10% of diagnosed TC. Sporadic and familial MTC accounts for 5% of thyroid cancers and originates from C cells. Hürthle cell carcinoma is rare (about 3%). Anaplastic thyroid cancer (ATC) is undifferentiated cancer that derives from a follicular cell, sometimes developing from an existing PTC or FTC. It is the rarest form (less than 2%) but the most aggressive TC, rapidly spreading into the neck and other body parts. ATC is difficult to treat and has a bad prognosis [3,4].

AITDs mainly comprise Hashimoto’s thyroiditis (HT) and Graves’ disease (GD) and affect 5% of the population, with a greater prevalence in women. AITDs are associated with the production of autoantibodies targeting specific thyroid proteins such as thyroid peroxidase (TPOAb), thyroglobulin (TGAb) and thyrotropin receptor antibodies (TSHRAb). Those antibodies disturb thyroid hormone production leading to hypo- or hyperthyroidism. AITDs are complex immune disorders characterized by infiltrating dysfunctional T lymphocytes with abnormal cytokine secretion. Since AITDs incidence has increased in recent years, since they are a predisposition for other non-thyroid autoimmune diseases, and since they significantly affect the quality of life, it is essential to identify early diagnostic markers and effective therapeutic targets [5].

## 2. Extracellular Vesicles (EVs)

### 2.1. Definition & Biogenesis

**Figure 2:**
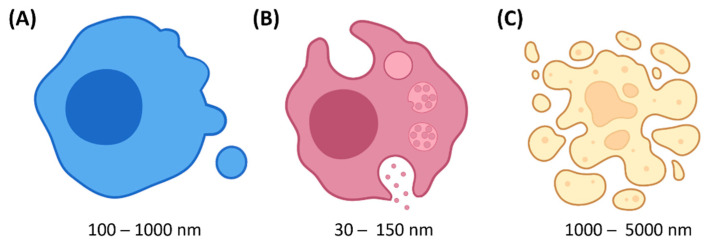
Schematic representation of the production mode of the different types of EVs. (**A**) Microvesicles are produced by direct budding at the plasma membrane. (**B**) Exosomes are released by exocytosis of a multivesicular body itself formed by the inward budding of a late endosome. (**C**) Apoptotic bodies are fragments of dying cells. Schemes are not to scale.

Extracellular vesicles (EVs) are a broad term describing non-replicating subcellular structures that are delimited by a lipid bilayer and released by cells. It includes microvesicles that directly bud outward from the plasma membrane, exosomes that derive from the endocytic compartment and are released following the fusion of multivesicular bodies with the plasma membrane, and apoptotic bodies that fragment from cells dying by apoptosis (Figure 2). Although EVs vary in size, one should not try to classify EV subtypes solely based on this parameter. Indeed, subtypes display overlapping sizes. EVs contain and protect a complex cargo of molecules. The spherical bilayered lipid membrane, in which membranous proteins are inserted, delineates a lumen encompassing soluble components such as cytosolic proteins, nucleic acids and metabolites. In the case of exosomes and microvesicles, diverse packaging mechanisms exist, some of which provide specificity to the loading of certain molecules. Consequently, EVs consist of a highly heterogeneous population with a large diversity in size, density, surface markers and enclosed cargo. EVs are produced by all cell types using ESCRT-dependent and -independent pathways and are present in all body fluids [6,7,8].

### 2.2. Function

EVs are considered a new communication paradigm used to transfer functionally active biological molecules from one cell to another [9]. Indeed, their complex and specific cargo recapitulates information about the producing cell and can induce functional responses in the recipient cell [10]. On the one hand, EVs have been shown to exhibit a plethora of physiological roles [7]. On the other hand, EV production and content are deregulated in pathological conditions and could therefore influence disease progression. In cancer, tumor-derived EVs have been reported to carry mainly detrimental functions, participating in the development and maintenance of a tumor-promoting microenvironment [11], but also promoting metastatic niche development at a distance [12]. They are also implicated in the regulation of immune processes and T-cell dysfunction [13].

### 2.3. Therapeutic and Diagnostic Potentials

Over time, and considering their role in disease progression, EVs and EV machineries have become a potential novel class of therapeutic targets. Moreover, their presence and stability in body fluids make them good biomarker candidates in liquid biopsies. Their cargo can indeed inform about the identity but also the state of the producing cell. Interestingly, the lipid, the protein, and the nucleic acid cargo of isolated EVs could be informative, and the combination of these markers could be the key to enhancing the specificity and sensitivity of the diagnostic [14]. Finally, EVs offer exciting opportunities to develop therapeutic vehicles for drug delivery. 

Isolated and unmodified EVs were shown to have positive effects on various pathological conditions, with the advantages of low immunogenicity, low toxicity and high stability [15]. Of course, the possibility of engineering EVs to deliver specific content (including protein and/or RNA) or to reach a specific target cell type further enhance the therapeutic opportunities brought by EVs. Despite the great promises offered by the EV field, technical issues, inter-laboratory standardization, and knowledge gaps about fundamental questions are still impairing the breakthrough of EVs in the clinic. One of the aims of the International Society for Extracellular Vesicles (ISEV) is to overcome those issues, particularly by publishing experimental requirements specific to the EV field [16] (update ongoing at the time of writing).

## 3. EVs in Thyroid Physiology

The first study that refers to EVs in the thyroid describes the ultrastructural changes that occur in the bat thyroid gland during arousal from hibernation. Using electron microscopy, Nunez et al. observed the appearance of small extracellular vesicles lying in the colloid close to the cell’s apical plasma membrane domain just before the emergence from hibernation. This EV release was preceded by an increase in the number of apical vacuoles and multivesicular bodies. Authors postulated that EV accumulation at the cell–colloid interface allowed for increased peroxidase production and extracellular iodination, thus increasing thyroid hormone production required upon arousal from hibernation [16]. A similar observation of ‘cell-surface shedding’ was made later in the thyroid gland of rats, mice and humans [17,18,19]. Intraluminal vesicles and dense luminal bodies were visualized in normal follicles and goiter progression. Vesicles ranged between 30 and 300 nm, and their numbers increased under thyroid hyperstimulation. These vesicles contain a membrane-bound peroxidase and possess iodinating capacity [18]. These structures are presumably derived from microvilli shedding of thyrocyte’s apical plasma membrane and were also detected in hyperactive human thyroid tissues [19]. Those structures could be a posteriori and described as microvesicles. More recently, Vlasov et al. demonstrated that FRTL-5 rat thyroid cells produced EVs containing undegraded TG. This process could represent an alternative pathway of TG processing, contrasting with lysosomal degradation and thyroid hormone production [20]. Despite these studies, the contribution of EVs to thyroglobulin iodination and thyroid hormone production has not been deciphered.

Thyroid parenchyma contains follicles surrounded by a complex stroma composed of abundant blood vessels, fibroblasts, C cells and resident immune cells [1]. Recently, Rosa et al. highlighted the existence of telocytes in normal human thyroid tissues, extending their cytoplasmic processes in the interfollicular spaces and around small blood vessels. Transmission electron microscopy images revealed the frequent presence of EVs surroundings the telopodes between thyrocytes, endothelial cells and monocytes [21]. The cellular origin of those EVs has not been elucidated. However, they could participate in thyroid tissue homeostasis, plasticity and synchronized adaptation of follicles to face the daily variations in iodine supply and T3/T4 needs. Indeed, angio-follicular units are isolated and independent factories for hormone production with different morphologies and functionalities. Various self-regulatory autocrine-paracrine signals are in place to regulate follicular activity. For example, follicles constantly and strictly control the adjacent microvasculature via TSH-independent ROS/HIF/VEGF pathways [1]. To what extent EVs could mediate those communications in a healthy thyroid is unknown. Our group demonstrated abundant endothelial and immune cell-derived EV populations in EV isolated from adult mouse thyroid lobes [22]. During thyroid development, endothelial progenitor cell (EPC)-derived EVs were shown to stimulate folliculogenesis of embryonic thyroid lobes cultured on filters by promoting thyrocytes organization and lumen expansion [23], which could be one way for endothelial cells to promote thyroid embryogenesis [24]. Therefore, the EV-mediated crosstalk between the different cell populations composing the thyroid parenchyma is possible in vivo and should be further explored.

## 4. EVs in TC

### 4.1. Role

EV function in TC has been mainly assessed using cell lines treated with EV isolated from culture media conditioned by thyroid cancer cell lines (TPC-1, BCPAP, CAL62, K1) compared to normal thyroid cell line (Nthy-ori 3-1) or from plasma/serum of patients. Functions were often attributed to specific constituents of the EV cargo: proteins, miRNAs, lncRNAs or circRNAs. However, in most cases, the presence of a specific cargo was simply correlated with tumorigenic progression without irrefutable evidence that the observed effect is effectively due to the identified molecular cargo. Those studies are listed in Table 1. Notably, the isolation method has an impact on the type of EV harvested and consequently on the results obtained; the dose used in in vitro experiments was rarely reported, as well as contamination by non-EV components. Moreover, only a few studies have investigated a specific cargo’s functional role or analyzed those effects in vivo.

### 4.2. TC-Derived EV-Mediated Role in Thyroid Cell Transformation

TC onset relies on the gain of malignant traits by transformed thyrocytes. Ultimately, increased migration and invasion capabilities characterize more aggressive and metastatic tumors. EVs have been implicated in the transfer of malignant properties from tumor cells to normal cells. For example, Annexin A1 (ANXA1), a well-known oncogenic protein overexpressed in TC tissues, has been shown to be transferred, via EVs, from the malignant SW579 cell line to the non-malignant Nthy-ori 3-1 thyroid cell line, thereby promoting proliferation, invasion and EMT of Nthy-ori 3-1 cells [25]. Acquisition of these properties depends on TGF-β1/Smad2 modulation and is confirmed in a xenograft mouse model [25]. Another study compared the effect of EVs isolated from 8305C, a human ATC cell line, and Nthy-ori 3-1, the non-malignant thyroid cell line, on recipient cells. ATC-EVs, but not Nthy-ori 3-1-EVs, increased cell proliferation and motility [26]. Furthermore, lncRNA CDKN2B-AS1 contained in cancer stem cell-like cells-derived EVs stabilized CDKN2B and promoted viability, migration, invasion and EMT of TPC-1 and SW579 cells via TGF-β1/Smad2/3 signaling pathway [27].

The LC-MS/MS proteomic analysis of EVs isolated from the serum of a pool of healthy controls and of patients with PTC associated or not with lymph node metastasis (LNM) revealed an increased abundance of proteins of the integrin family (ITGA2, ITGA2B, ITGAV, ITGB1, ITGB2, ITGB3) and of proteins involved in metastasis (TLN1, ITGB2, CAPNS1, SRC) in EVs from PTC patients with LNM. Those EVs promoted BHT101 cancer cell invasiveness in vitro without any effect on cell migration [28]. Besides these studies highlighting the role of EVs in malignancy, it is important to mention that contrasting results were also recently reported. The effect of EVs isolated from different thyroid cancer cell lines (CGTH, FTC-133, 8505c, TPC-1 and BCPAP) was assessed on Nthy-ori 3.1 without demonstrating any significant effect on proliferation, migration and invasion [29].

The transfer of malignant traits via TC-derived EV was also attributed to different RNA species carried by EVs. EVs derived from TPC-1, a PTC cell line, contain a greater abundance of miR-146b and miR-222 as compared to EVs from Nthy-ori 3-1 cells. Although TPC-1 EVs, but not Nthy-ori 3-1 EVs, inhibited Nthy-ori 3-1 cell proliferation, it was not demonstrated to be miRNA-dependent [30]. TPC-1 undergoing EMT was shown to contain the lncRNA MALAT1 and the EMT effectors SLUG and SOX2. The treatment of TPC-1 cells in a normal state with EMT-related EVs enhanced MALAT1 expression as well as EMT and stem-like cell markers without affecting invasiveness. Thyroid cancer stem-like cell-derived EVs transport linc-ROR in addition to MALAT1 and functionally transfer those molecules, thereby causing an increase in the proliferative and invasive capabilities of Nthy-ori 3-1 [31]. SNHG9, a lncRNA overexpressed in PTC tissues compared to adjacent thyroid specimens, is also overexpressed in the PTC cell lines, TPC-1 and K1, as compared to Nthy-ori 3-1. It was shown that SNHG9 is enriched in EVs from PTC cells and that the EV-mediated transfer of SNHG9 inhibited autophagy and promoted apoptosis of Nthy-ori 3-1 through interaction and negative regulation of YBOX3 and P21 [32].

### 4.3. TC-Derived EV-Mediated Communication with the Tumor Microenvironment

Thyroid cell transformation is accompanied by the remodeling of thyroid tissue and changes in cell populations within the microenvironment. Those changes can be triggered by cancer cell-derived EVs and cause the reprogramming of EV-mediated communications within the tissue. Our group demonstrated an increase in EVs derived from epithelial and immune cells within the thyroid microenvironment after BRAF^V600E^ induction in a PTC mouse model. While abundant recruitment of immune cells in the transformed thyroid could explain the increase in immune cell-derived EVs, it is not the case for the epithelial compartment. Based on the miRNAs content of EVs, we postulated that epithelial EVs could support the establishment of a permissive immune microenvironment [22]. Along the same line, a study on pediatric thyroid cancer reported a higher level of PD-1 and PD-L1 on EVs in children with PTC as compared to healthy children, correlating with clinical features. In addition, treatment of CD8+ T-cells with PD-L1 EVs decreased the expression of inflammatory cytokines, thus interfering with effector T-cell activation and promoting immune escape [33].

Furthermore, proteomic analysis performed on EVs isolated from the human ATC cell line, 8305C, and normal thyroid cell line, Nthy-ori 3-1 revealed increased diversity of proteins in ATC-EVs, with proteins enriched in immune-related pathways [26]. Proteins implicated in integrin signaling, tumor-promoting pathways, neutrophil degranulation and angiogenesis were also more abundant in ATC-EVs [26]. Functionally, cancer-derived EVs negatively affected HUVEC tube formation but did not change monocyte phagocytosis [29]. The crosstalk between tumoral or normal thyroid cells (TPC-1, 8505c, Nthy-oi 3-1) and fibroblasts was studied in co-culture experiments. CD147+EVs released by fibroblast-thyroid tumor cell co-cultures induced the expression and release of metalloproteinases (MMP2/9) from normal fibroblasts, enhancing the migratory phenotype of tumor cells [34]. CD147, initially known as EMMPRIN (extracellular matrix metalloproteinase inducer), is a glycoprotein overexpressed in many types of cancers and associated with a poor prognosis. The same group recently confirmed the regulation of extracellular matrix remodeling by EVs isolated from thyroid cancer cell-fibroblast co-culture [35], illustrating EVs’ dynamic role.

In a PTC mouse model, we showed that the miRNA content of EVs followed the miRNA deregulation found in thyroid tissue upon BRAF^V600E^ induction. In EVs, as in the tissue, we detected an early upregulation of miRNAs with oncogenic properties and a delayed or secondary downregulation of miRNAs with tumor suppressor activities. Changes in the abundance of the more abundant EV-miRNAs were mainly due to cancer cell-derived EVs and were consistent with observations from human thyroid diseases. KEGG pathways analysis revealed that those EV-miRNAs were enriched in immune-related pathways [22]. The transfer of miRNAs from cancer cells to cells of the microenvironment via EVs has been studied by several groups in vitro. MiR-181a is abundantly found in EVs derived from the plasma of a patient with PTC and FTC, and PTC cell lines (BCPAP, K1), and this abundance is even more evident when cells are cultured under hypoxic conditions [36,40].

Interestingly, miR-181a delivery to HUVECs via EVs promoted cell proliferation and the formation of a capillary-like network. In vivo, this miRNA also induced angiogenesis and tumor growth. Mechanistically, it was shown that miR-181a affected the MLL3/DACT2/YAP-VEGF axis [36]. A similar effect of EVs isolated from hypoxic thyroid cells on HUVEC angiogenesis has been demonstrated to depend on the presence of miR-21-5p in EVs. In that case, miR-21-5p directly targeted and suppressed TGFB1 and COL4A1, thereby increasing endothelial tube formation. Interestingly, EV derived from the sera of PTC patients exerted a similar effect on cultured HUVEC [37]. Identifying EV-miRNA targets and studying miRNA-mRNA networks in a global and unbiased manner greatly benefited from the development of bioinformatics tools. A comparison of the profile of EV-miRNAs released by two thyroid cancer cell lines with that of non-cancerous thyroid cells revealed a differential abundance of five miRNAs (miR-21-5p, miR-31-5p, miR-221-3p, miR-222-3p, and let-7i-3p) in EVs of tumor cells, with three of them (miR-31-5p, miR-222-3p, and let-7i-3p) being more abundant in EVs from the more aggressive cancer cells [41]. These miRNAs were also present in EVs isolated from thyroid tissue after BRAF^V600E^ induction [22]. Functional enrichment and network analysis of these miRNAs’ target genes (ICAM1, FOXO3, SELE, ETS1, RECK, PTEN, and TIMP3) are regulators of these miRNAs from aggressive thyroid cancer cells of the tumor microenvironment through modulation of angiogenesis and immune response [41].

### 4.4. Link with BRAF and Resistance to Treatment

TC is a genetically simple disease with a relatively low somatic mutation burden in each tumor, and the type of cancer will depend on the driver mutation [3]. The most frequent genetic alterations occur in *BRAF*, *RAS* and *RET* genes. These genes encode the BRAF, RAS and RET proteins, both acting upstream of the MAPK (Mitogen-Activated Protein Kinase)/ERK (Extracellular signal-Regulated Kinase) pathway. This pathway controls cell proliferation and is over-activated in about 30% of all cancers. The most frequent mutation in the *BRAF* gene is a T1799A transversion that substitutes valine residue with a glutamate residue in the protein sequence (noted V600E) [3,42].

A few studies link BRAF^V600E^ mutation and MAPK signaling pathway activation with the deregulation of EV release and content. Inhibition of the constitutively activated MAPK signaling pathway in cancer thyroid cell lines triggered an increase in the number of EVs released per cell. Conversely, the same inhibition in normal thyroid cells did not affect EV production [43]. It is worth mentioning that several studies performed on melanoma, which also harbor the BRAF^V600E^ mutation, and whose observations could potentially be extended to TC. Inhibition of BRAF with vemurafenib significantly increased EVs’ total RNA and protein content and caused significant changes in the RNA profiles loaded in EVs. In particular, an abundance of the miR-211-5p level was increased. This is interesting since miR-211-5p transfection reduces cell sensitivity to vemurafenib in target cells [44]. Another group studied the role of EVs in developing resistance to BRAF inhibition in melanoma cells. They showed that PDGFRb could be transferred via EVs from resistant melanoma cells to recipient melanoma cells, resulting in a dose-dependent activation of PI3K/AKT signaling and escape, thus resistance, from BRAF inhibition [39]. Finally, EVs produced by a melanoma cell line resistant to BRAF inhibition was also shown to carry increased levels of PD-L1 [45]. Altogether, these studies underline the link between EVs, BRAF^V600E^ and BRAF-inhibitor resistance in melanoma while stressing the need for additional functional and in vivo studies as well as an extension to BRAF^V600E^-dependent TC.

### 4.5. Diagnosis

A challenging issue in the TC field is the differential diagnosis between malignant tumors and benign nodules. The diagnosis of TC is mainly based on the interpretation of fine-needle aspiration biopsy (FNAB) utilizing the six-tiered Bethesda System for Reporting Thyroid Pathology (BSRTC), with each category associated with a risk of malignancy. However, FNAB is invasive, non-compatible with treatment follow-up and often inconclusive. Indeed, the increase in TC incidence appears to be the result of the misclassification of benign nodules in the malignant groups and the detection of cancers with pathological criteria for malignancy that do not lead to symptoms or death. This overdiagnosis inevitably leads to overtreatment, which causes, in TC, unnecessary surgeries [46]. More accurate diagnostic tests and non-invasive biomarkers are thus needed to discriminate between TC and benign thyroid diseases.

Several groups evaluated the potency of EVs in liquid biopsy to assist TC detection, prognosis and monitoring (Table 2). Thyroperoxydase (TPO) was recently found at the surface of thyroid-derived EV circulating in the blood. Furthermore, it was shown that miRNA let-7 enclosed in those TPO+ EV could distinguish between FTC and follicular adenomas with high accuracy [47]. Thyroglobulin was also detected in EVs derived from rat thyroid cells [20] and in urinary EVs isolated from patients with PTC and FTC [48]. Other proteins have been proposed as potential biomarkers for clinical applications. Indeed, levels of HSP27, HSP60, and HSP90 were higher in EVs isolated from the plasma of PTC patients as compared to patients with benign goiter, thereby reflecting differences observed in tissue.

Furthermore, their levels were reduced after ablative surgery [49]. The comparison of the proteome profiles of serum-derived EVs from PTC patients with or without lymph node metastases (LNM), and healthy donors, identified a set of deregulated proteins associated with cancer cell metastasis. The upregulation of integrins, integrin-associated proteins and chaperones (HSP27) in EV from patients with LNM could therefore be considered diagnostic and prognostic markers [28].

Several studies have addressed the utility of circulating EV-miRNAs as biomarkers in TC and highlighted different candidates. A first stimulating report revealed that miR-21 and miR-181a could potentially differentiate a PTC from a FTC with great sensitivity and specificity [40]. Furthermore, lower levels of EV-enclosed miR-130 and miR-29a were associated with worse clinical variables [38,50]. A three EV-miRNA signature (miR-346, miR-10a-5p and miR-34a-5p) was also identified for PTC diagnosis in Chinese patients [51]. In another study, six EV-miRNAs (miR-16-2-3p, miR-223-5p, miR-34c-5p, miR-182-5p, miR-223-3p, and miR-146b-5p) isolated from plasma had a significantly lower abundance in a patient with nodules as compared to healthy individuals [52].

On the contrary, miR-16-2-3p and miR-223-5p were more abundant in PTC patients. Receiver operating characteristic (ROC) analyses revealed that combined miRNA panels showed increased diagnostic sensitivities and specificities compared to single miRNA markers [52]. A recent in silico study used sequencing data in the TGCA database to identify a different signature, composed of 6 miRNAs, comprising EV-enriched miR-129-2 and miR-889 as predictors of PTC prognosis [53]. We measured the levels of 6 miRNA candidates in tissue, in total plasma and highly purified plasma-EVs. Only miR-146b-5p and miR-21a-5p displayed a significant differential abundance in EVs purified from the plasma of patients with PTC as compared to benign disease. No difference could be demonstrated in bulk plasma, and no correlation could be highlighted between abundance in EVs and expression in tissue [54]. The absence of a relationship between the abundance of most free and encapsulated miRNA in the serum of a patient with thyroid diseases was also reported [55].

Several studies assessed prognosis markers for lymph node metastases (LNM). A first study described higher levels of miR-146b-5p and miR-222-3p in PTC patients with LNM compared to those without LNM [56]. In another study, two other EV-miRNAs, miR-6774-3p and miR-6879-5p, were proposed as biomarkers for the prognosis of LNM in PTC patients [57]. In the third one, four miRNAs (miR24-3p, miR146a-5p, miR181a-5p and miR382-5p) were identified as differentially secreted in EVs from PTC patients without correlation to the presence of LNM [55].

Other EV-enclosed RNA species (circRNA, lncRNAs) have been proposed as biomarkers. CircRNA has been demonstrated to play a role in TC progression and to be associated with certain clinicopathological features [58]. Yang et al. compared the circRNA content of serum-EVs isolated from a patient with PTC or MNG. High-throughput sequencing, validated by RT-qPCR, identified 22 circRNAs with altered levels in PTC patients [59].

Strikingly, small overlaps were found between those studies, highlighting the heterogeneity and the current lack of reproducibility in EV-based biomarker research. Two groups used next-generation sequencing to identify miRNA candidates presenting a differential abundance in plasma-derived EVs from patients with PTC or with benign nodules [60,61]. Even though those two studies had a comparable design and number of patients, they came out with different discriminating miRNAs for malignant and benign diseases. This clearly illustrates discrepant results between studies with the same goal.

### 4.6. Treatment

Treatment of a patient with suspected TC requires a surgical procedure to altogether remove the primary lesion (thyroidectomy or lobectomy). Radioactive iodine treatment (RAI, iodine-131), external radiotherapy, chemotherapy, and targeted therapies such as tyrosine kinase inhibitors are other treatments that can be prescribed alone or in combination. Radioactive iodine treatment requires the expression of the sodium iodide symporter (NIS) at the basal pole of thyrocytes. However, in advanced and dedifferentiated TC, such as ATC, thyroid-transformed cells lose the expression of NIS and become resistant to radioactive iodine therapy. Several strategies are investigated to recover NIS expression and restore iodine avidity in cancer cells. The treatment with tyrosine kinase inhibitors can reach this. EVs isolated from primary human adipose-derived stem cells (ADSCs) have been used as delivery vehicles for TKIs since they can be efficiently loaded and taken up by radioactive iodine-refractory thyroid cancer cells (SW1736). Compared to the free-TKI treatment, EVs^TKI^ were more efficient in increasing the expression levels of iodide-metabolizing mRNAs and proteins and enhancing ^125^I uptake in SW1736 cells, therefore re-establishing radioiodine-sensitivity in vitro [62]. With the same goal in mind, EVs were used for the direct transfer of NIS protein to hepatocellular carcinoma (HCC) cells. EVs isolated from NIS-transfected HCC cells were shown to contain NIS protein (but not mRNA) and to effectively transfer the iodide transporter to recipient cells, consequently enhancing the ^125^I uptake and ^131^I cytotoxicity [63,64].

Other strategies in TC treatment consist in targeting molecular pathways controlling tumorigenesis mechanisms such as cell proliferation, migration or metabolism. The inhibition of those pathways can be achieved by small non-coding RNAs such as miRNAs and siRNAs. EVs isolated from human umbilical cord mesenchymal stem cells and containing miR-30c-5p were used to target PELI1, a ubiquitin ligase aberrantly expressed in PTC and known to promote cell proliferation and invasion via the PI3K-AKT pathway. Treatment with miR-30c-5p-EVs decreased the PELI1 mRNA and protein expression in PTC (W3) cells, as well as their proliferation and migration. These data were confirmed in a xenograft model by demonstrating tumor growth inhibition upon intra-tumoral injection of miR-30c-5p-EVs [65]. In another study, cancer cell fatty acid anabolism was targeted via siRNA-mediated inhibition of stearoyl-CoA desaturase-1 (SCD-1). Two ATC cell lines treated with SCD-1 siRNA-EVs showed decreased viability caused by ROS accumulation [66].

TCs are considered inflammatory and immunogenic tumors. The density and type of immune infiltrate (tumor-infiltrating leukocytes, tumor-associated macrophages) correlate with the clinical outcome of TC patients [67,68]. Therefore, EV-based immune cell reprogramming and immunotherapy are also valuable therapeutic opportunities in TC. For example, NK-cell-derived EVs were shown to display antitumor activity in different models, including thyroid cancer [69]. Moreover, priming NK cells with IL-15 increased EV content in granzyme B, perforin, and FasL and enhanced EV cytotoxicity against ATC cells (CAL62) without affecting normal thyroid cells [70]. All these promising in vitro results need to be extended and confirmed in vivo settings.

## 5. EVs in AITD

### 5.1. Role

Although the pathogenesis of AITDs is still not clear, this group of diseases is caused by the presence of autoantibodies deregulating the physiology of the gland. In GD, TSHR autoantibodies are produced and constitutively stimulate thyroid hormone synthesis via TSHR activation and cAMP signaling. Interestingly, TSHR was detected in EVs produced by normal and tumoral thyroid cells [71]. It was thus proposed that AITD could be caused by antibodies reacting against TSHR-bearing EVs [72]. On the other hand, Edo et al. suggested that TSHR-bearing EVs are rather beneficial for the patient since these TSHR-EVs were able to bind and sequester TSHR autoantibodies, exerting a decoy effect and improve thyroid function [71].

AITDs are characterized by an abnormal immune response with an important role in T-cell reprogramming. Here, the role of EVs in the induction of inflammatory and immune responses in AITD was investigated. First, it was reported that circulating EVs from HT patients harbored more TPO, HSP60 and MHC-II than circulating EVs from healthy controls. These EVs were taken up by peripheral blood mononuclear cells (PBMCs), particularly CD14+ monocytes and CD11c+ dendritic cells (DCs). As a consequence of TLR activation, activated DCs stimulated a strong CD4+ T lymphocyte response, potentially contributing to HT onset. Moreover, HSP60, whose structural conformation is similar to TPO and TG, could serve as an antigen for autoantibody production [73]. The same group, later on, showed that EVs from IFN-γ-treated Nthy-ori 3-1 thyroid cells also harbored TPO, HSP60 and MHC-II and promoted DC activation (increase of CD40, CD80, CD83, IL-6 and TNF) which caused a switch in CD4+ T cells toward a pro-inflammatory phenotype [74]. Finally, they showed that circulating EVs from GD patients transported more IGF-1R and HSP60 than EVs from healthy controls. Those GD-EVs could bind to TLR2/3 on PBMCs and initiate a pro-inflammatory response characterized by an increase in IL-6 and IL-1β production [75]. Circulating AITD-EVs was also shown to inhibit Treg differentiation and promote T CD4+ activation and T helper 17 differentiation in vitro [76]. Furthermore, EVs isolated from the tear fluid of GD patients with eye disease were more abundant and displayed different protein content (increased VDB, CRP, CHI3L1, MMP-9 and VCAM-1), as compared to control tears-EVs. In addition, the exposition of orbital fibroblasts to those EVs enhanced the production of IL-6, IL-8 and MCP-1, thereby activating inflammatory responses [77]. Finally, circulating EVs from resistant GD patients stimulated IL-1β and TNF-α expression in PBMCs as compared to EVs from GD patients in remission or healthy controls [78]. Altogether, these studies indicate that AITD-EVs can activate DCs and trigger T-cell reprogramming associated with an imbalance in cytokine expression and release, possibly involved in systemic inflammation. These studies are reported in Table 3. Of note, none of these studies has proved the role of the identified cargo in the observed effect. Furthermore, it is important to stress that the EVs isolation methods used (differential ultracentrifugation and commercial kits) do not avoid the co-isolation of contaminants. Further investigations are required in order to confirm an EV-dependent effect.

### 5.2. Diagnosis

The number and origin of circulating EVs in blood circulation varied with AITD. Indeed, the number of circulating EVs derived from platelets, monocytes and endothelial cells was increased in the blood of GD patients as compared to healthy controls. This increase could be due to cell apoptosis caused by the activation of inflammatory reactions and reflect monocyte activation. In addition, an increase in E-selectin- and VE-cadherin-positive EVs was measured in GD blood samples, suggesting endothelial dysfunction. Interestingly, the increase in EV number was reduced by thiamazole anti-thyroid therapy [79]. In another study, quantification of circulating EVs in plasma from AITD patients and healthy patients revealed a higher percentage of platelet-derived EVs (CD41a+) in AITD and lower percentages of leukocyte- and endothelial cell-derived EVs [76]. The changes in the percentage of circulating EV populations in the blood may be a sign of systemic involvement in AITDs.

Differences in EV content have been searched to be more accurate and specific to the disease state (Table 4). For example, and as mentioned, differences were found in the levels of TPO, HSP60 and MHC-II in circulating EVs from HT patients [73] or of IGF-1R and HSP60 in circulating EVs from GD patients [75] as compared to healthy individuals. Comparison of the protein content of circulating EVs from patients with GD, HT and healthy controls provided a list of proteins, differentially abundant in EVs and mainly enriched in the immune system and or involved in metabolism [80]. Analysis of miRNA cargo also revealed potential specific biomarkers. MiR-146a and miR-155 levels were increased in circulating EVs isolated from patients with AITD and have been involved in innate and adaptative immunity processes [76]. Another study revealed an increase of circulating miR-23b-5p and miR-92a-39, and a decrease of let-7g-3p and miR-339-5p in GD patients in remission as compared to those with incurable GD [78]. Despite the fact that those observations were made using total serum, i.e., without EV purification, these 4 miRNAs have already been detected in blood-EVs using deep-sequencing [81]. Finally, an analysis of circRNA levels in circulating EVs from GD patients and healthy controls by microarray and RT-qPCR revealed an increased presence of CircRNA_000102 in patients with GD [82].

### 5.3. Treatment

The therapeutic utility of EVs in AITD has not been exploited yet. However, the miRNA content of mesenchymal stem cell-derived EVs has been proposed as an ideal immunomodulatory therapy for multiple autoimmune diseases, including rheumatoid arthritis, autoimmune hepatitis, colitis and systemic lupus erythematosus [83]. This proposal could be transposed and extended to AITDs. Additionally, the function of TSHR-EV should be further investigated in order to conclude unequivocally on the beneficial or adverse effect of these TSHR-EVs in AITD pathogenesis [71,72]. If a decoy role is confirmed, TSHR-EVs could prevent autoantibody-mediated activation of thyroid function [71]. In another therapeutic approach, artificial phosphatidylcholine vesicles fabricated by microfluidic technology have been proposed as delivery vehicles for triiodothyronine (T3). Liposomal formulation of thyroid hormones improved the delivery and accumulation of hormones in target cells [84]. The ability of EVs to encapsulate and transport T3/T4 has not yet been studied.

## 6. Concerns & Further Directions

### 6.1. EVs Production & Release

The biogenesis and distribution of thyrocyte-derived EVs should be elucidated in the healthy and diseased states to evaluate their potential pathophysiological role and, eventually, their target cells. As in every polarized epithelial cell, EVs could be secreted at the apical and/or the basolateral pole. Released at the apical pole, their circulating capacities inside the dense proteinaceous colloid matrix and their capture by adjacent thyrocytes are questionable. The capacity of EVs to leave the colloid wherein they have been produced, e.g., by para-cellular route or transcytosis, is unknown. EVs could indeed be internalized by the extremely active endocytic system of thyroid cells and then either degraded, recycled, unboxed or released at the other side of the cell. Another possible function for apically-released EV would be to increase the density of membrane-bound proteins involved in iodine processing in contact with the colloid. Released at the basal pole, EVs should first cross the basal lamina in order to envision an eventual EV-dependent cross-talk between follicles and other cells. To confirm EV-mediated crosstalk inside the thyroid microenvironment, it would be interesting to undoubtedly demonstrate the transfer of EVs from thyroid (cancer) cells to other cell populations. Genetically-engineered mouse model allowing the expression of fluorescently-tagged tetraspanins (CD9, CD81, CD63) under the thyroglobulin promoter could help to decipher the biogenesis mechanism(s) of thyrocyte-EVs in normal and transformed conditions, but also to visualize and identify their target cells if they exist. Since the unique structure of the thyroid is lost during tumorigenesis, EV distribution and movements within the tissue may be totally different in cancer tissue as compared to normal tissue. For example, in the case of cancer cells, the basal lamina may be fragilized or partially destroyed, thereby facilitating EVs movement away from the transformed thyrocytes. Fluorescent EVs could also be useful for detecting and following EVs in circulation. Indeed, once released in interfollicular spaces, EVs could reach blood circulation through the dense network of fenestrated capillaries. A recent study confirmed the capacity of thyroid EVs to reach circulation by detecting TPO-positive EVs in the blood [47]. TG precursor was also detected in EVs from bovine serum [85].

### 6.2. EVs Function

EV function has mainly been studied in in vitro settings, i.e., in cultured cells. However, the monocellular 2D culture of cells spreading and growing on plastic dishes is far from physiological conditions. First, the phenotype and genotype of isolated and cultured cells can evolve from the original tumor. Indeed, thyroid cancer cell lines display a mRNA expression profile that is closer to poorly differentiated/dedifferentiated cancers than to differentiated papillary thyroid cancer [86]. 

Secondly, the absence of 3D organization and of cellular heterogeneity clearly reduces the complexity of biological mechanisms to their simplest form. Local and systemic intercellular exchanges, cell co-dependency, microenvironmental selective pressure, and interaction with and circulation across the extracellular matrix are phenomena that do not operate in those simple 2D in vitro models. 

For future studies, the development of physiologically relevant in vitro and ex vivo models would be of great interest. For example, some researchers have recently demonstrated the ability of a microfluidic device to maintain human Graves’ disease tissue enabling the isolation of Graves’ disease-specific EVs [87]. Others described a method of 3D rat thyroid follicles culture in Matrigel that reproduces physiological follicle structure and activity [88]. Finally, advances in generating thyroid organoids could also help and be inspiring for thyroid-derived EV functional studies [89,90]. A recent review also highlighted the possibility of boosting EV production using a 3D culture system, among other possibilities [91]. Finally, excessive dosages with questionable physiological relevance are often used in in vitro assays [92,93].

### 6.3. Diagnosis

The goal of new biomarkers discovery is to better identify or classify pathological conditions to reduce overdiagnosis and overtreatment, i.e., unnecessary surgeries. Overtreatment is due to uncertain diagnosis of malignant nodules but also to excessive clinical importance given to indolent microcarcinomas [94,95]. Indeed, in most cases, low-risk thyroid cancer will never impact a patient’s life. Active surveillance has been proposed as an appropriate management strategy for those TC [96,97]. New guidelines will probably favor TC monitoring rather than the precautionary principle that was largely in place until now. New, accurate, specific, and non-invasive biomarkers, coupled with rational management of thyroid nodule cases, will reduce the number of unnecessary surgeries.

The discovery of EV-based biomarkers in diseases, including thyroid diseases, suffers from important discrepancies between studies. Logistical and technical difficulties due to the low quantity of circulating components, lack of reproducibility and absence of consensus about the most suitable protocol for EV purification can be proposed as explanations, but also highlight the need for standardization of pre-analytical and analytical procedures [98]. Currently, those parameters and others, such as the cost and the feasibility of EV purification techniques, still hinder the clinical application of EV and particularly in EV-miRNAs detection from blood biopsies [99,100]. Furthermore, the impact of individual variability on blood EV count and content is totally unknown and could significantly impact both inter- and intra-patient variability [101,102,103]. To overcome this high variability, studies on a large cohort of patients must be conducted to evaluate sensitivity and specificity and ultimately validate biomarker candidates [103].

The major difficulty of using EVs as a biomarker is the scarce abundance of tissue-specific EVs mixed with all-organ EVs in circulation. The detection of a cancer-derived EV is considered a rare event in plasma from a patient with small-to-medium-size-tumor, probably even rarer in the case of small, indolent or encapsulated carcinomas such as PTC. Therefore, the enrichment of EV preparation with thyroid-specific EVs should be considered in future studies. An essential prerequisite is the identification of thyroid-specific markers on the EV surface. Immunocapture of thyroid/cancer-derived EV in microfluidic devices could then allow the analysis of the content of this pure or at least enriched disease-related EV population.

### 6.4. Treatment

The use of EVs as vehicles for drug delivery is progressing and largely explored to treat a variety of diseased conditions. Nevertheless, there is still work to do in order to fill the gaps in our understanding of EV biology. Unraveling the molecules and mechanisms governing cargo loading, target cell specificity and content delivery, as well as the rules controlling the EV journey in vivo, may ultimately allow scientists to exploit the knowledge about EV and increase the efficacy of therapeutic EV production and targeting specificity. The current ineffective loading and delivery of cargo are indeed key issues. In addition, the therapeutic development of EV will also benefit from the improvement of current EV isolation methods to reach time- and cost-effectiveness [104]. This is essential for the large-scale production of good manufacturing practice (GMP)-grade EVs [105].

Importantly, none of the aforementioned therapeutically-oriented studies assessed the efficacy of the proposed treatment in orthotopic tumors. Studies on the biodistribution of EVs in mice and macaques did not show accumulation in the thyroid [106,107]. Those studies have never been conducted in the presence of thyroid cancer which could redirect EV uptake. Indeed, EV distribution could shift toward inflamed zones. Using a new bioluminescent reporter system, the systemic injection of ATC cell (CAL62)-derived EVs in mice effectively showed a tropism to subcutaneous CAL62 tumor [108]. However, the subcutaneous tumor could be more accessible than a thyroid cancer in its protected environment. Moreover, the homing capacity of those CAL62-derived EVs could be due to their recognition of parental CAL-62 cells. Considering the harmful effect (increased proliferation, invasion, etc.) of tumor-derived EVs, their use as therapeutic agents should not be recommended. In that context, the reported beneficial effect of mesenchymal stem cell-derived EVs may be a good alternative.

EVs delivery to target tumor cells should be demonstrated and optimized. Specific targeting, improved fusion capacity and enhanced stability can be theoretically achieved by modifying the EV surface. The goal would be to redirect the biodistribution of EVs that would normally accumulate in the filtering organs such as the liver, kidneys and spleen when administrated to animal models [109]. This would first potentiate EV effects by increasing bioavailability but also limit off-target effects and toxicity. It is thus essential to identify thyroid cancer cell- or immune cell-specific receptors from which ligands could be selected for incorporation into the EV membrane or to identify homing peptides.

## Figures and Tables

**Table 1:** List of publications analyzing the function of EVs in TC. Nthy—Nthy-ori 3-1; (d) UC—(differential) Ultra-Centrifugation; PEG—PolyEthylene Glycol; DG—Density Gradient; OE—OverExpression; KD—Knock Down; ab—antibody.

Reference	EV Isolation Technique	Identified Content	EV Origin	Recipient Cells	Dose	Effects	In Vivo?	Link between Content and Effect
(Li et al., 2021) [25]	Kit	ANXA1	SW579, Nthy-ori3-1	SW579, Nthy-ori3-1	?	Malignant transformation	Xenograft	OE, KD
(Surman et al., 2022) [26]	dUC	Proteins	8305C, Nthy-ori3-1	8305C, Nthy-ori3-1	30–60 µg/1 × 10^4^ cells or /well	Viability, migration	/	/
(Wu et al., 2022) [27]	dUC	Cdkn2b, Cdkn2b-AS1	Nthy-ori3-1, SW579	TPC-1, SW579	?	Viability, migration, invasion	/	OE, KD
(Luo et al., 2018) [28]	dUC	SRC, TLN1, ITGB2 and CAPNS1	PTC serum	BCPAP, BHT101	30–100 µg/3 × 10^4^ cells	Invasion	/	/
(Grzanka et al., 2022) [29]	ExoQuick	/	Nthy-ori3-1, FTC6133, CGTH-W-1, BCPAP, 8505C, TPC-1	Nthy-ori3-1, HUVEC, PBMC	?	No effect on Nthy-ori3-1 and monocytes, ↘tube formation by HUVEC	/	/
(Lee et al., 2015) [30]	dUC	miR-146b, miR-222	TPC-1, Nthy-ori3-1	TPC-1, Nthy-ori3-1	?	Inhibition of proliferation	/	/
(Hardin et al., 2018) [31]	ExoQuick	lncRNA	TPC-1, THJ-16T, Nthy-ori3-1	TPC-1, Nthy-ori3-1	5–10 µg/1 × 10^5^ cells or 5 mg/1 × 10^4^ cells	Proliferation, invasion	/	/
(Wen et al., 2021a) [32]	PEG	SNHG9 lncRNA	TPC-1, K-1, Nthy-ori3-1	Nthy-ori3-1	?/co-culture	Autophagy, apoptosis	/	OE, KD
(Delcorte et al., 2022b) [22]	dUC+DG	miRNAs	CTL and BRAF^V600E^ thyroid tissues	BMDM	2 × 10^3^ EVs/cells	BMDM polarization	/	/
(Wang et al., 2020) [33]	Kit	PD-L1	PTC plasma	Activated T cells from PBMCs	?	Immunosuppression	/	Anti-PD-L1 ab
(Bravo-Miana et al., 2020) (Bravo-Miana et al., 2022) [34,35]	dUC	Proteins	Fb, 8505C, TPC-1, Nthy-ori3-1/co-culture	Fb, 8505C, TPC-1, Nthy-ori3-1	?	MMP2 activation	/	/
(Wang et al., 2021) [36]	dUC, ExoQuick	miR-181a	BCPAP, K-1, Nthy-ori3-1	HUVEC	?	Tube formation	Xenograft	anti-miR-181a
(Wu F, 2019) [37]	dUC	miR-21-5p	BCPAP, KTC-1, Nthy-ori3-1	HUVEC	?	Angiogenesis	/	anti-miR-21-5p
(Yin et al., 2021) [38]	Kit	miR-130a-3p	DTC plasma	TPC-1	?	↗IGF-1, p-PI3K, p-AKT and migration	/	siRNA anti-Igf1
(Vella et al., 2017) [39]	dUC+DG	PDGFRb	Melanoma cell lines	Melanoma cell lines	50–200 µg/mL on 4 × 10^5^ cells	Resistance to treatment	/	neutralizing PDGFRβ ab

**Table 2:** List of publications questioning the diagnostic value of EVs in TC. HC—Healthy Controls; BN—Benign Nodules; MNG—Multinodular Goiter; PTC—Papillary Thyroid Cancer; N0/N1—without/with lymph node metastases; (d) UC—(differential) Ultracentrifugation; IDC—Iodixanol Density Cushion; SEC—Size Exclusion Chromatography; DG—Density Gradient; NGS—Next-Generation Sequencing; qRT-PCR—quantitative Real-Time Polymerase Chain Reaction; DEs—Differentially Expressed.

Reference	Initial Material	Patients	EV Isolation Technique	Content Analysis	Differential Content
(Zabegina et al., 2020) [47]	Plasma	30 FTC, 30 FA	dUC, immuno-capture	qRT-PCR	let-7
(Huang et al., 2020) [48]	Urine	16 PTC, FTC	Kit	LC-MRM/MS	Thyroglobulin
(Caruso Bavisotto et al., 2019) [49]	Plasma	13 PTC, 18 MNG	dUC	WB	HSP27, HSP60, HSP90
(Luo et al., 2018) [28]	Serum	16 PTC-N1, 17 PTC-N0	dUC	LC-MS/MS	SRC, TLN1, ITGB2 and CAPNS1
(Samsonov, 2016) [40]	Plasma	60 PTC, FTC, BN	dUC	qRT-PCR	miR-21, miR-181a
(Wen et al., 2021b) [50]	Serum	119 PTC, 100 HC	ExoQuick	qRT-PCR	miR-29a
(Yin et al., 2021) [38]	Plasma	40 DTC, 40 BN	Kit	qRT-PCR	miR-130a-3p
(Wang et al., 2020) [51]	Plasma	43 PTC	Kit	ELISA	PD-L1
(Liang, 2020) [52]	Plasma	51 PTC, 38 MNG	Kit	NGS	6 miRNA signature
(Xin et al., 2022) [53]	TGCA data	491 PTC	/	/	6 miRNA signature
(Delcorte et al., 2022a) [54]	Plasma	19 MNG, 17 PTC	IDC, SEC, UF	qRT-PCR	miR-146b-5p, miR-21a-5p
(Capriglione F, 2021) [55]	Serum	56 PTC-N1, 58 PTC-N0	ExoQuick	miRNA array cards	miR24-3p, miR146a-5p, miR181a-5p and miR382-5p
(Jiang et al., 2020) [56]	Plasma	49 PTC-N1, 15 PTC-N0	Kit	qRT-PCR	miR-146b-5p, miR-222-3p
(Chen et al., 2022) [57]	Plasma	34 PTC-N1, 34 PTC-N0	dUC	microarray	miR-6774-3p, miR-6879-5p
(Yang et al., 2019) [59]	Serum	3 PTC, 3 MNG	Kit	RNA seq	CircRNAs
(Pan et al., 2019) [60]	Plasma	13 PTC, 7 MNG	dUC, DG	NGS	126 DE miRNAs
(Dai et al., 2020) [61]	Serum	136 PTC, 92 MNG, 51 HC	dUC	small RNA seq	miR-485-3p, miR-4433a-5p

**Table 3:** List of publications analyzing the function of EVs in AITDs. HT—Hashimoto’s Thyroiditis; GD—Graves’ Disease; DC—Dendritic Cell; PBMC—Peripheral Blood Mononuclear Cell; (d)UC—(differential) UltraCentrifugation; WB—Western Blot; qRT-PCR—quantitative Real-Time Polymerase Chain Reaction. ↗ stands for “increase of”; ↘ stands for “decrease of”.

Reference	EV Isolation	Content Analysis	Differential Content	EV origin	Recipient Cells	Dose	Effects
(Cui et al., 2019) [73]	High-speed dUC	WB	TPO, HSP60, MHC-II	HT serum	DCs, PBMCs	600 µg- EVs/1 × 10^6^ cells	Action via TLR2/3 DCs: ↗IL-6, NF-kB PBMCs: Th1, Th17, Treg diff.
(Cui et al., 2020) [74]	High-speed dUC	WB	TPO, HSP60, MHC-II	IFN-γ-treated Nthy	DCs	Transwell co-culture	DC activation, CD4+ T ly pro-inflammatory response
(Cui et al., 2020) [75]	High-speed dUC	WB	IGF-1R, HSP60	GD serum	PBMCs	400 µg-EVs/2 × 10^6^ cells	Action via TLR2/3 ↗IL-6, IL-1b NF-Kb pathway
(Rodríguez-Muñoz et al.) [76]	Medium-speed UC	qRT-PCR	miR-146a, miR-155	AITD plasma	Naive CD4+ T cells	1mL plasma-MVs/2 × 10^5^ cells	↗ Th17 and ↘Treg differentiation
(Han et al., 2021) [77]	Kit	Proteome profiler	VBD, CRP, CHI3L1, MMP-9, VCAM-1	GD tear fluids	Orbital fibroblasts	5 µg/1 × 10^4^ cells	↗ IL-6, IL-8, MCP-1
(Hiratsuka et al., 2016) [78]	Kit	/	/	GD serum	PBMCs	?	↗ expression of TNF-a, IL-1b, IL-6

**Table 4:** List of publications questioning the diagnostic value of EVs in AITDs. HC—Healthy Controls; HT—Hashimoto’s Thyroiditis; GD—Graves’ Disease; (d) UC—(differential) UltraCentrifugation; WB—Western Blot; qRT-PCR—quantitative Real-Time Polymerase Chain Reaction; DEcRs—Differentially Expressed circRNAs.

Reference	Initial Material	Patients	EV Isolation Technique	Content Analysis	Findings
(Cui et al., 2019) [73]	Serum	30 HT, 30 HC	High-speed dUC	WB	TPO, HSP60, MHCII: HT > HC. No difference in TG, HMGB1, ICAM1
(Cui et al., 2021) [75]	Serum	33 GD, HC	High-speed dUC	WB	IGF-1R, HSP60: GD > HC
(Jia et al., 2021) [80]	Plasma	12 GD, 10 HT, 7 HC	High-speed dUC	Proteomics	List of differential proteins implicated in the immune and metabolic systems
(Rodríguez-Muñoz et al.) [76]	Plasma	33 GD, 29 HT, 45 HC	Medium-speed UC	qRT-PCR	Mir-146a, miR-155: AITD > HC
(Sun et al., 2020) [82]	Plasma	25 GD, 25 HC	High-speed dUC	CircRNA microarray	15 DEcRs hsa_circRNA_000102: GD > HC

## Data Availability

Not applicable.
